# Age-related differences in muscular coordination while performing elbow flexion and extension movements at different velocities

**DOI:** 10.1038/s41598-025-19126-z

**Published:** 2025-09-12

**Authors:** Elisa Romero Avila, Hannah Lena Siebers, L. Cornelius Bollheimer, Catherine Disselhorst-Klug

**Affiliations:** 1 Department of Rehabilitation & Prevention Engineering, Institute of Applied Medical Engineering, RWTH Aachen University, Pauwelsstr. 20, 52074 Aachen, Germany; 2 Experimental Orthopaedics and Trauma Surgery Teaching and Research Area, RWTH Aachen University, Pauwelsstr. 30, 52074 Aachen, Germany; 3 Department of Geriatric Medicine, RWTH Aachen University, Pauwelsstr. 30, 52074 Aachen, Germany

**Keywords:** Aging, Motor control, Muscular coactivation, Upper limb, Electromyography, Healthy aging, Skeletal muscle, Orthopaedics, Geriatrics

## Abstract

Life expectancy is increasing worldwide, with older adults expected to account for 16% of the population within 30 years. Understanding age-related declines in muscle function is crucial for prevention and maintaining independence in Activities of Daily Living (ADL). Additionally, a major consequence of these declines is the development of the physical frailty phenotype. Therefore, this study aimed to identify changes in muscular coordination during elbow flexion and extension with aging and varying movement velocities. Twelve older adults (76 ± 6.5 years) performed the movements at varying angular velocities using a constant 1 kg external load (0.4 Nm). Elbow joint kinematics and muscle activity (biceps brachii, brachioradialis, and triceps brachii) were recorded using motion capture and surface electromyography and compared to 15 healthy young adults (26.2 ± 3.2 years) under identical conditions. Clinical assessments in the older group also evaluated comorbidities, ADL dependency, and frailty risk. Despite being considered healthy, older adults showed indicators of physical decline and exhibited increased muscular coactivation, possibly as a compensatory mechanism to maintain joint stability. However, coordination patterns remained unchanged across velocities, indicating stability with age. These findings suggest changes may occur before clinical decline and should be considered in training strategies for older adults.

**Supplementary Information:**

The online version contains supplementary material available at 10.1038/s41598-025-19126-z.

## Introduction

According to the World Health Organization, there is a notable increase in life expectancy worldwide^1^. People over 60 years of age are expected to account for 16% of the global population in less than thirty years. Moreover, within the same period, the population of people over 80 years of age is expected to be three times higher than the current figures. However, data suggests that this increase in life expectancy is not directly associated with an increase in well-being as there are declines in both physical, cognitive, and mental health as people age^1^.

Referring to physical changes, there is an age-related loss in muscle mass and strength, decline in proprioception function, and increased muscle stiffness that contribute to an elevated risk of injuries, disability and overall reduction in the quality of life^2–5^. Yet, these changes appear to be muscle-specific, varying depending on the analyzed muscle, its function, and its characteristics^6,7^. The alterations in muscle mass, strength, and function observed in people over 65 years old further influence the force-velocity relationship. An age-related decrement of muscle force is observed at all angular velocities and types of contraction as well as a decrease in the muscle contraction velocity^8,9^. However, these changes are more pronounced during concentric force, as eccentric force appears to be better preserved in older adults^8^.

A negative consequence of these age-related physical changes in terms of loss of muscle mass and strength is the development of frailty. There is an increased prevalence of frailty as people get older, especially in women, as close to 25% of people over 65 years old are frail^10,11^. Frailty, according to Fried´s physical phenotype (PFP), encompasses several symptoms associated with less resistance to stress and decreased function of different organs^12^. A person will be considered frail if it has three or more symptoms and pre-frail if it meets one or two symptoms. As can be expected, an older person with frailty has an increased risk of injuries, disability, and, ultimately, death^10,13^. However, adjustments in the person’s lifestyle can worsen, remain stable, or reverse frailty^10^.

To compensate for the previously mentioned age-related limitations and be able to execute and control a movement, older adults rely on the Central Nervous System (CNS) adopting different control strategies^14^. Once such strategy is increased and prolonged muscular coactivation to improve stability^15–20^. Muscular coactivation involves the simultaneous increased activation of the synergistic and antagonistic muscles, which represents an increment in metabolic energy that leads to fatigue, reduced flexibility, increased stiffness, and may also contribute to developing joint diseases^16–18,21^.

Particularly on the upper extremities, older adults have demonstrated increased muscular coactivation compared to young adults in various studies^22–24^. Analyzing these changes in upper extremity muscles is relevant because, compared to young adults, these muscles have an important role in maintaining independence during Activities of Daily Living (ADL)^11,25^. For example, older adults may use their arms to assist when standing up from a chair.

The aim of this work is to investigate age-related differences in the muscular coordination of the biceps brachii, brachioradialis and triceps brachii during elbow flexion and extension performed at various movement velocities, with the goal of understanding the control strategies adopted by the CNS to perform movements despite age-related changes. Additionally, the influence of frailty symptoms on muscular activation across these velocities will be evaluated. Elbow flexion/extension was selected because it is essential for performing ADLs, and allows most of the muscles involved to be recorded using surface electromyography (sEMG), thus enabling a more accurate evaluation of the coordination patterns^14^.

We hypothesize that older adults will exhibit increased muscular coactivation than young adults across all angular velocity categories due to age-related alterations in muscle function and coordination. Furthermore, we expect that a higher number of frailty symptoms in older adults will be associated with higher muscular coactivation compared to young adults, although this is considered a secondary aim to explore possible tendencies related to frailty.

## Methods

### Participants

Twelve subjects (six female, six male; average age of 74.18 ± 4.2 years) were recruited at the Clinic for Geriatric Medicine of the RWTH Aachen University Hospital, Germany. Inclusion criteria considered participants over 65 years, right-hand dominant (self-reported), and mentally able to follow the protocol, according to the Mini-Mental Test (score above 27 points). Individuals were excluded if they had upper limb disorders, visual impairments, experienced pain at the time of the measurements, or met more than three criteria of the Fried Frailty Phenotype (i.e. a diagnosis of frailty). All participants provided written informed consent, and the local ethics committee of the medical department at RWTH Aachen University approved the protocol (EK 346/19).

### Clinical assessments

Besides the “Mini-Mental Test” and the “Fried Frailty Phenotype”, three additional scales were implemented to evaluate the health status and functional abilities of the older adults group: the “de Morton Mobility Index (DEMMI)”, the “Barthel Index for Activities of Daily Living (ADL)”, and the “Charlson Comorbidity Index (CCI)”. These tests help identify the ability of older people to execute ADLs and predict the risk of mortality based on comorbidities.

### Instrumentation

Subjects were asked to perform flexion and extension of the elbow with an added load of 1 kg, selected to provide minimal resistance to the movement. For young adults, previous work by von Werder et al. has shown that loads below 10% of the maximal voluntary contraction (MVC) torque help avoid fatigue and altered coordination patterns associated with heavier loads^26^. For older adults, previous studies have recommended the use of light external loads to minimize fatigue and preserve typical control strategies^27–29^.

**Fig. 1:**
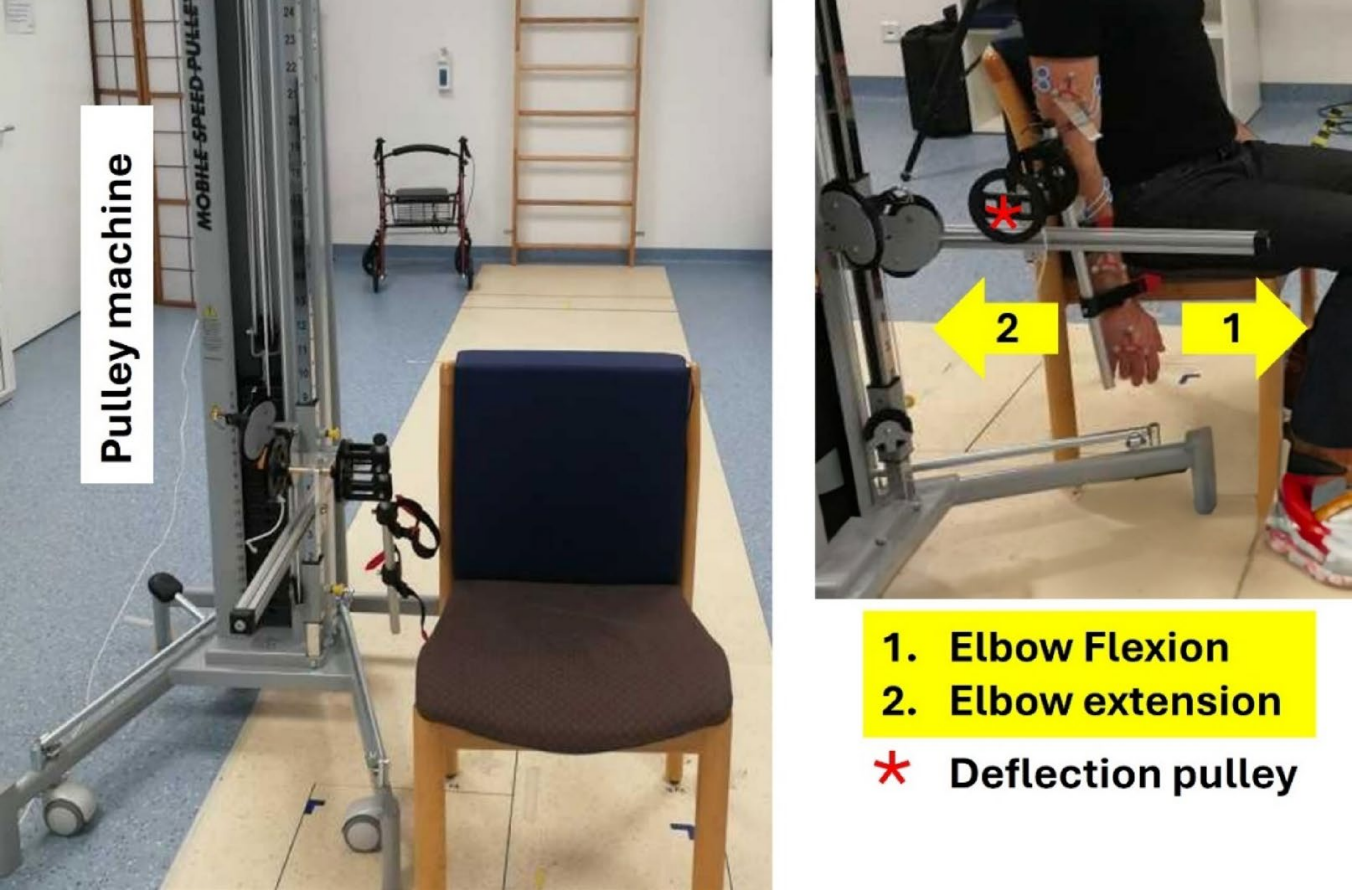
Pulley machine used for the measurements. The picture on the right shows a subject connected to the pulley machine through the deflection pulley.

Subjects were seated next to a standard pulley machine equipped with a deflection pulley (radius: 4 cm) to which the subjects’ forearms were connected, generating an external torque of approximately 0.4 Nm (Fig. 1). This setup ensured constant external torque throughout the full range of flexion/extension elbow movements. Since the height could be adapted, the elbow coincided with the middle of the deflection pulley, and the shoulders relaxed in a neutral position.

### Protocol

Once the subjects were fastened to the pulley machine, they flexed and extended the elbow at different angular velocities (ranging from 20°/s to 140°/s) for 45 s, followed by a 1-minute rest period. This series was repeated three times, producing more than 20 repetitions of flexion and extension elbow movements. To perform the movement, participants were guided by a feedback system that presented velocity patterns (20°/s to 140°/s) in a randomized order^26^. They were instructed to match their movement to these patterns, while the actual angular velocity was computed from the kinematic data.

The velocity ranges were based on a categorized analysis approach of von Werder et al., which addresses the challenges of sEMG analysis during dynamic movements by dividing the range of angular velocities into intervals^26^. This allows for comparisons across subjects while ensuring sufficient samples per category.

### Data gathering and analysis

Muscular activation of the biceps brachii, brachioradialis, and triceps brachii was recorded during the elbow flexion and extension movements using a bipolar sEMG configuration with pre-gelled electrodes (Ambu, Germany) and a conventional system (Noraxon, USA). The electrodes were positioned based on the recommendations from the project on *surface EMG for non-invasive assessment of muscles* (SENIAM) for biceps and triceps brachii and on the muscle belly of the brachioradialis^30,31^. Inter-electrode distance was defined at 20 mm, and the subjects’ skin was prepared before the measurements. This included cleaning the skin with alcohol and waiting for it to dry completely before placing the electrodes^30^. The sEMG signals were then recorded with a sampling rate of 1500 Hz, 500 gain, bandpass filtering between 10 and 500 Hz, and a 16-bit A/D converter. During post-processing, a separate bandpass Butterworth filter (10–500 Hz) was applied to the signal before rectification and smoothing with a moving root mean square (RMS) window of 500 ms to obtain the sEMG envelope. The resulting sEMG envelope was normalized by dividing it by a reference value calculated for each subject, muscle and movement phase (flexion or extension). This reference was obtained from the maximum values of the signals for each condition, arranged in descending order, and the average of the upper half of these values was taken.

To capture the elbow flexion and extension movements, a motion capture system (Qualisys AB, Göteborg, Sweden) was used at a sampling rate of 200 Hz, and an upper-body biomechanical model was considered^32^. The elbow’s kinematics were computed using this model, and the elbow flexion/extension angle signal was filtered using a Butterworth low-pass filter (2nd order, cutoff frequency 2 Hz). Using a modified version of a decision tree algorithm originally developed by von Werder et al., time-normalized sEMG envelopes were arranged into eight categories based on the elbow’s kinematics^33^. The first two categories refer to the phases of elbow flexion and extension, followed by four angular velocity categories: 20–40°/s, 40–60°/s, 60–100°/s, and 100–140°/s. The corresponding sEMG envelope values were then averaged separately for each category.

A preexisting database from von Werder et al. containing measurements from 15 healthy young adults (7 male, 8 female, age 26.2 years ± 3.2) under identical conditions was incorporated into the study^26^. The sEMG data from this database was categorized using the previously described algorithm. This data was then compared to the one collected from the older adults to evaluate age-related changes in muscular activation.

Following the comparison between the two age groups, the database of the older adults was further divided based on their frailty score to explore possible differences in muscular coordination patterns related to frailty. The “Frailty score 0” (FS0) group included older adults with no symptoms of frailty, while the “Frailty score 1” (FS1) group consisted of those at intermediate risk of frailty with 1 or 2 symptoms.

### Statistical analysis

To evaluate statistically significant age-related differences in muscular activation across angular velocity categories, two separate analyses of variance (ANOVA) were conducted using SPSS (IBM Corp, NY). The first ANOVA examined the effects of age on the muscular activation of the three muscles across different angular velocity categories. The second one assessed the differences in muscular activation based on the frailty score. For each comparison, the outcome measure was the mean value of the normalized sEMG envelope for each muscle, obtained for each movement phase and angular velocity category. Assumptions of normality and homogeneity of variance-covariance matrices were verified, and the significance level was defined at α = 0.05. Post-hoc analyses were performed using the Least Significant Difference (LSD) test where needed, and Cohen’s d was calculated to measure effect size.

## Results

Table 1 shows the clinical assessments results. According to the DEMMI score, all subjects had independent mobility (scores between 62 and 100). However, *Subject 4* had a moderate dependency, while *Subject 7* had a slight dependency for performing ADLs, as Barthel Index scores between 61 and 90 indicate moderate dependency and 91–99 indicate slight dependency. *Subject 4*, with moderate dependency in ADLs, had a 52% chance of mortality within one year (CCI score of 4). Additionally, around 60% of the subjects, including *Subject 4*, were at an intermediate risk of frailty (Frailty Score 1 or 2). Handgrip strength, measured with a dynamometer as part of the frailty assessment, was below the German reference thresholds for low muscle strength in two participants (*Subject 4* and *Subject 9*)^34^.

**Table 1:** Results of the clinical assessments for older adults: de Morton mobility index (DEMMI), Barthel index for activities of daily living (ADL), Charlson comorbidity index (CCI), and fried frailty phenotype (with handgrip strength). These scales are validated for their use on people over 65 to assess their health status and functional abilities.

Subject	Assessment				
	DEMMI	Barthel	CCI	Frailty	Handgrip strength (kg)
Subject 1	100	100	0	0	41.47
Subject 2	100	100	2	0	27.53
Subject 3	100	100	2	1	22.4
Subject 4	100	80	4	1	25.7
Subject 5	100	100	0	1	25.27
Subject 6	100	100	1	0	21.77
Subject 7	100	95	0	0	25.53
Subject 8	100	100	0	1	28.6
Subject 9	100	100	1	0	31.4
Subject 10	100	100	1	1	41.5
Subject 11	100	100	0	2	27.86
Subject 12	100	100	0	1	32.5

**Fig. 2:**
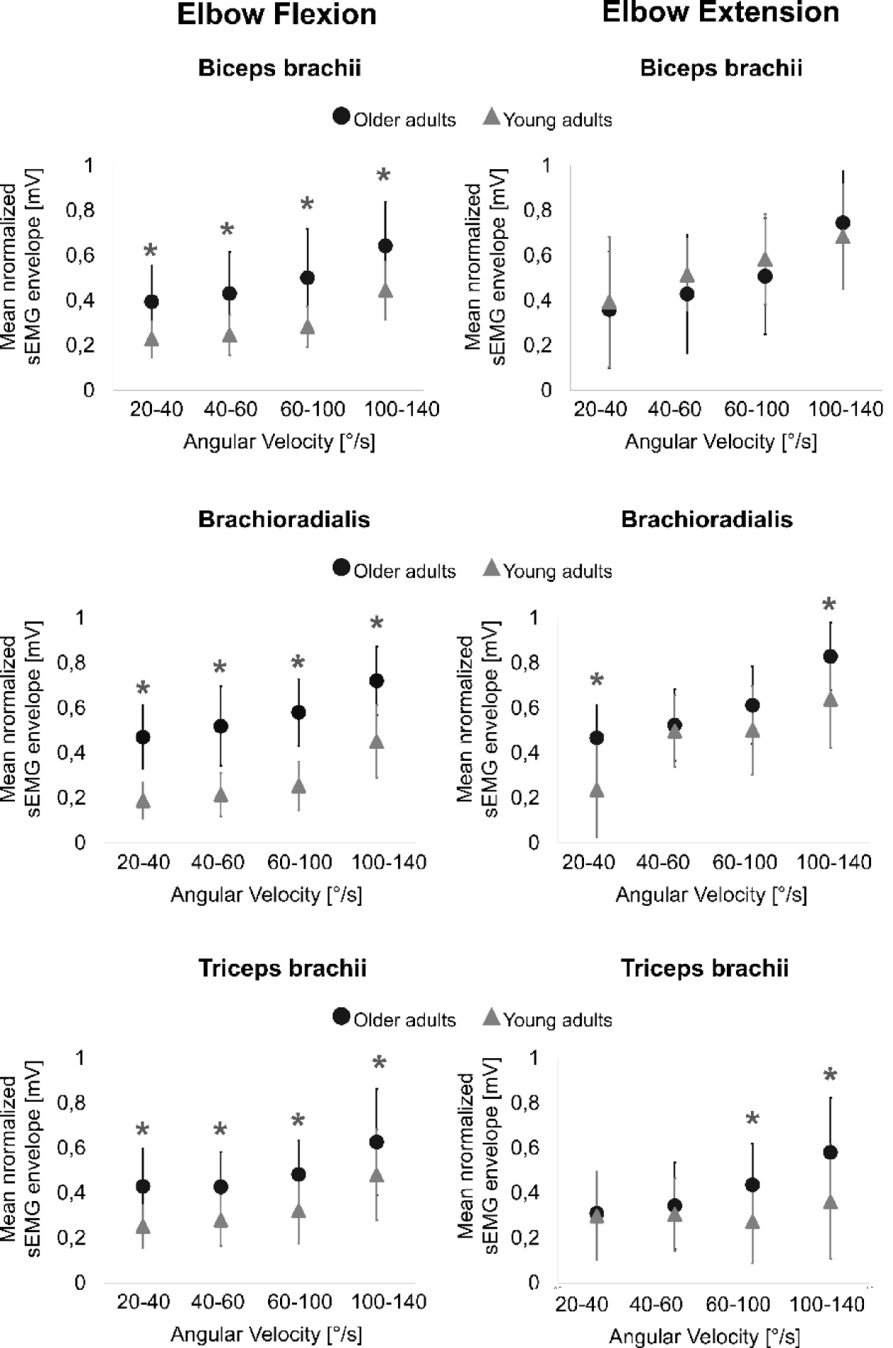
Mean normalized sEMG envelope of the biceps brachii, brachioradialis, and triceps brachii during elbow flexion and extension at four angular velocity categories: 20–40°/s, 40–60°/s, 60–100°/s, and 100–140°/s. ● shows the values of the old adults and ▲ those of the young adults. Significant differences between young and older adults are indicated with *.

Muscular activation of the biceps brachii, brachioradialis, and triceps brachii was significantly higher in older adults compared to younger subjects during elbow flexion across all angular velocity categories (*p* < 0.001; Fig. 2; Table 2). Additionally, the effect sizes were large for all muscles and angular velocities during elbow flexion (Cohen’s d > 0.8; Table 3), except for the triceps brachii at the fastest angular velocity category, where the effect size was medium (Cohen’s d = 0.676; Table 3).

During elbow extension, age-related differences in muscular activation were statistically significant in the brachioradialis at the slowest and fastest angular velocity categories (*p* < 0.001), and in the triceps brachii between 60–140°/s (*p* < 0.018). The effect sizes were also large in these specific cases (Cohen’s d > 0.844; Table 3). Significant differences were also found between the slowest (20–40°/s) and fastest (100–140°/s) angular velocity categories for all muscles in both age groups (*p* < 0.01), except for the triceps brachii in young adults during elbow extension (see Supplementary Table S1 online). Notably, the interaction between age and angular velocity categories was not significant for any muscle (*p* > 0.1), indicating that the changes in muscular activation across angular velocity categories did not differ with age.

**Table 2:** Mean (and standard deviation) of the normalized sEMG envelope of the biceps brachii, brachioradialis, and triceps brachii of young and older adults across angular velocity categories during elbow flexion and extension. ANOVA results are also presented for angular velocity categories and age group comparisons. Significant differences are indicated with *.

Mean normalized sEMG envelope (standard deviation)									ANOVA		
Angular velocity categories	20–40 °/s		40–60 °/s		60–100 °/s		100–140°/s		p-value velocity	p-value group	p-interaction
Group	Young	Old	Young	Old	Young	Old	Young	Old			
Flexion											
Biceps brachii	0.22 (0.08)	0.39 (0.16)	0.24 (0.08)	0.43 (0.18)	0.28 (0.09)	0.5 (0.21)	0.44 (0.12)	0.64 (0.19)	< 0.001*	< 0.001*	0.935
Brachioradialis	0.18 (0.08)	0.47 (0.14)	0.21 (0.09)	0.51 (0.17)	0.25 (0.1)	0.58 (0.14)	0.45 (0.16)	0.72 (0.15)	< 0.001*	< 0.001*	0.880
Triceps brachii	0.25 (0.09)	0.42 (0.16)	0.27 (0.11)	0.42 (0.15)	0.32 (0.14)	0.48 (0.15)	0.47 (0.2)	0.62 (0.23)	< 0.001*	< 0.001*	0.985
Extension											
Biceps brachii	0.39 (0.29)	0.35 (0.25)	0.51 (0.16)	0.42 (0.26)	0.58 (0.2)	0.5 (0.25)	0.68 (0.23)	0.74 (0.22)	< 0.001*	0.519	0.680
Brachioradialis	0.23 (0.21)	0.46 (0.14)	0.49 (0.15)	0.52 (0.15)	0.5 (0.19)	0.61 (0.17)	0.63 (0.21)	0.82 (0.14)	< 0.001*	< 0.001*	0.265
Triceps brachii	0.31 (0.18)	0.30 (0.16)	0.31 (0.17)	0.34 (0.19)	0.27 (0.19)	0.43 (0.18)	0.34 (0.25)	0.58 (0.24)	0.068	0.018*	0.167

**Table 3:** Effect sizes (Cohen’s d) for muscular activation differences between young and older adults in the biceps brachii, brachioradialis and triceps brachii during elbow flexion and extension. Effect size values and their corresponding descriptions are provided.

Angular velocity categories	Effect size (Cohen’s D) and descriptions							
	20–40 °/s		40–60 °/s		60–100 °/s		100–140°/s	
Elbow Flexion								
Biceps brachii	1.337	Large	1.306	Large	1.343	Large	1.235	Large
Brachioradialis	2.497	Large	2.193	Large	2.536	Large	1.721	Large
Triceps brachii	1.327	Large	1.108	Large	1.098	Large	0.676	Medium
Elbow Extension								
Biceps brachii	−0.124	Small	−0.385	Small	−0.328	Small	0.268	Small
Brachioradialis	1.373	Large	0.165	Small	0.597	Medium	1.016	Large
Triceps brachii	−0.027	Small	0.136	Small	0.844	Large	0.961	Large

Following the analysis between the young and older adults, the differences according to the frailty score in older adults were assessed. Table 4 presents the mean normalized sEMG envelope of the three muscles for older adults with no frailty symptoms (FS0) and intermediate frailty risk (FS1); corresponding values for young adults are reported in Table 2. ANOVA results comparing the three groups (young adults, FS0, and FS1) are also included in Table 4. Although significant differences were found between the groups and across angular velocity categories for all muscles during elbow flexion and extension (*p* < 0.001 for flexion and *p* < 0.04 for extension), the interaction between groups and angular velocity categories was not significant (*p* > 0.5), suggesting that the changes in muscular activation across angular velocity categories did not differ between young adults and older adults with different frailty symptoms.

**Table 4:** Mean (and standard deviation) of the normalized sEMG envelope of the biceps brachii, brachioradialis, and triceps brachii of older adults with no frailty symptoms (FS0) and intermediate risk of frailty (FS1) across angular velocity categories during elbow flexion and extension. Corresponding values for young adults are reported in Table 2. ANOVA results are also presented for angular velocity categories and age group comparisons (Young, FS0 and FS1). Significant differences are indicated with *.

Mean normalized sEMG envelope (standard deviation)									ANOVA		
Angular Velocity Categories	20–40 °/s		40–60 °/s		60–100 °/s		100–140°/s		p-value velocity	p-value group (young, FS0 and FS1)	p-interaction
Group	FS0	FS1	FS0	FS1	FS0	FS1	FS0	FS1			
Flexion											
Biceps brachii	0.34 (0.08)	0.42 (0.19)	0.39 (0.13)	0.46 (0.22)	0.45 (0.23)	0.53 (0.21)	0.61 (0.18)	0.66 (0.2)	< 0.001*	< 0.001*	0.997
Brachioradialis	0.41 (0.05)	0.51 (0.17)	0.46 (0.08)	0.55 (0.21)	0.50 (0.04)	0.63 (0.17)	0.66 (0.12)	0.76 (0.16)	< 0.001*	< 0.001*	0.997
Triceps brachii	0.49 (0.11)	0.38 (0.19)	0.47 (0.05)	0.39 (0.19)	0.46 (0.01)	0.49 (0.2)	0.68 (0.08)	0.58 (0.3)	< 0.001*	< 0.001*	0.971
Extension											
Biceps brachii	0.21 (0.15)	0.46 (0.27)	0.28 (0.18)	0.53 (0.26)	0.35 (0.18)	0.61 (0.25)	0.64 (0.27)	0.81 (0.17)	< 0.001*	0.002*	0.935
Brachioradialis	0.36 (0.1)	0.54 (0.12)	0.41 (0.1)	0.59 (0.15)	0.50 (0.12)	0.68 (0.16)	0.79 (0.14)	0.85 (0.15)	< 0.001*	< 0.001*	0.523
Triceps brachii	0.30 (0.13)	0.31 (0.2)	0.30 (0.16)	0.37 (0.21)	0.40 (0.11)	0.46 (0.22)	0.56 (0.26)	0.58 (0.25)	0.02*	0.049*	0.595

## Discussion

Research on aging is complex. On the one hand, although the older adults considered for this study were healthy and had independent mobility, they still showed declines related to age, and more than half were at intermediate risk for frailty. Additionally, the increase in the sEMG values observed in the older adults suggests that, although most of them can independently perform ADLs (as evaluated by the clinical assessments that include upper limb tasks such as feeding, bathing, dressing, and grooming), they still rely on higher muscular activation to compensate for the age-related declines and being able to perform even less complex tasks, such as elbow flexion and extension. This pattern indicates that the CNS adopted a control strategy identified by increased muscular coactivation, as there was a simultaneous increase in the activation of all muscles responsible for the movement and statistically significant differences between young and older adults. Increases in muscular coactivation have also been observed in adults under psychological stress, or when it is necessary to increase joint stiffness during unfamiliar movements^14,21^.

On the other hand, the interaction between age and angular velocity was not statistically significant, thus indicating that muscular coordination patterns remain unchanged in older adults. In fact, the preservation of muscular coordination patterns with age may be attributed to the frequent performance of this movement in ADLs, as suggested by Sun et al.^35^. This is further supported by previous findings reporting stable muscular coordination patterns during familiar and unfamiliar elbow tasks, regardless of movement velocity^14^.

Although limited, research has examined the interaction between age and movement velocity in muscular activation. In the lower extremities, Monaco et al. conducted a muscle synergy analysis and found consistencies between young and older adults in the synergy factors and weight coefficients during walking^36^. Our results align with those of Monaco, but in the context of upper extremity movements, as we didn’t find an interaction effect between age and movement velocity in muscular activation, suggesting that the movement was executed in a similar way in both groups. This further reinforces the idea that while muscular activation may change with age, coordination patterns remain stable.

Other studies have reported age-related changes in muscular coactivation of the elbow flexor and extensor muscles^22,37^. In those studies, coactivation was assessed as the ratio of agonist-antagonist pair, whereas in our study it was interpreted from the simultaneous increase in the sEMG values of all muscles responsible for the movement, together with the statistical differences observed between young and older adults. This interpretation aligns with the recommendation of Mohammadyari et al. to consider the term muscular coactivation when analyzing sEMG signals^38^. Both this approach and ratio-based methods have consistently indicated that aging is associated with increased activation of the muscles involved in the task^22,37^. Bautmans et al., reported increased biceps brachii coactivation during maximal isometric elbow extension in the older adults, as well as greater triceps brachii coactivation during maximal isometric elbow flexion in both young and older adults^22^. Similarly, Darling et al., observed higher coactivation of biceps and triceps brachii during elbow flexion and extension in older adults^37^.

Although increased coactivation aims to protect and stabilize a joint, it may also lead to limitations in fine motor control, earlier onset of fatigue, and reduced range of motion^6,39^. Given that many older adults in this study were already at intermediate risk of frailty, these changes may progressively increase their susceptibility. Previous studies have reported that increased antagonist activation can reduce agonist muscle activation, opposing its intended action and thereby reducing the joint’s maximal force production^22,37,40^. This reduction occurs because joint net torque results from the difference between agonist and antagonist muscle torques^21^. Furthermore, increased antagonist activation contributes to the age-related loss of muscle strength, along with changes in muscle fiber type distribution, increased non-contractile tissue within muscles, and alterations in tendon elasticity^41,42^. Regarding the fiber type distribution, there is a particular atrophy of type II fibers as people age that leads to a greater proportion of type I fibers, which are less powerful^43,44^. As a result, overall force generation is reduced, requiring greater muscular activation to compensate^43^. This compensation mechanism may support older adults to perform demanding tasks or movements at higher velocities. Consequently, both the reduction in net torque due to increased antagonist activation and the particular atrophy of type II fiber may explain the increased sEMG values observed in older adults. Moreover, the biceps brachii, which has a higher proportion of type II fibers, is particularly susceptible to these age-related changes^45^.

As a secondary analysis, the division of the older adults group based on the number of symptoms related to frailty showed that the higher the frailty score, the greater the muscular activation. Indeed, Theou et al. and Jones et al. observed differences in muscular activation across the biceps brachii, triceps brachii, vastus lateralis and biceps femoris in non-frail, pre-frail and frail older adults^11,25^. This suggests that the analysis of muscular activation can support clinical assessments in effectively identifying both early and intermediate risk of frailty, particularly when age-related changes – considered in this study - are also accounted for (e.g. differences between young adults and non-frail older adults – FS0). Another relevant finding is that no significant interaction effect between the number of frailty symptoms and angular velocity was observed, suggesting that muscular coordination remains consistent in non-frail and pre-frail subjects. Nevertheless, this secondary analysis was based on a small and unequal subgroup size (five older adults for FS0 and seven for FS1), which limits the statistical power. Therefore, these results should be interpreted as preliminary and replication in larger and more balanced cohorts is necessary to confirm these patterns. While the observed differences are consistent with previous reports, the current findings should be viewed as indicative of a possible trend, rather than conclusive results.

This study has certain limitations that should be considered when interpreting the results. The sample of older adults was small, and the subdivision according to the number of frailty symptoms was unequal. Together, these factors reduced the statistical Power to 65% (calculated with G*Power^46^) for detecting a medium effect size (f = 0.25) at *p* = 0.05 in the ANOVA. While a Power of 80% is typically preferred, 65% still offers a meaningful probability of detecting relevant effects, particularly given the consistent trends observed across multiple muscles and velocities. Despite its limitations, the results of the study are largely consistent with previous literature on age-related changes in muscular activation and provide valuable insights into motor control in older adults.

Some strategies to promote the maintaining of functional ability and well-being as one grows older (a term known as “healthy aging”) involve regular exercise^47–49^. Exercise, specifically resistance training, is regarded as effective in improving physical health in older adults^50^. Several authors agree that, despite age-related deteriorations, it is still possible for older adults to adapt in response to exercise^37,51,52^. Therefore, exercise programs should aim to enhance muscle strength, flexibility and balance, while also considering movement velocity and the age-related changes in motor control strategies adopted by the CNS, such as increased muscular coactivation, to better manage these adaptations^35^.

To our knowledge, this study is among the few that utilize clinical assessments, completed either by the subjects or testers, with quantitative methods, such as sEMG, to evaluate older adults, particularly in the upper extremities. By considering clinical and quantitative approaches, this study offers a broader perspective on physical decline in aging since the clinical assessments used in this study are widely recognized for evaluating health status and detecting indicators of any physical decline, while the complementary approach of sEMG provided objective insights into the muscular function. Additionally, the recording of the activation of most muscles responsible for the movement allowed for a detailed evaluation of the control strategies, revealing an age-related increase in muscular coactivation as angular velocity increased, while coordination patterns remained unchanged. This suggests that changes in muscular activation may occur even before detectable signs of physical deterioration appear on clinical scales^53^.

Given these results, an important direction for future research is the development of algorithms that consider both clinical scales and muscular activation for the early detection of physical declines in older adults. Moreover, these findings should be incorporated into the development of training strategies for older adults, ensuring that both compensatory mechanisms as well as preserved coordination patterns are considered when developing training strategies to prevent physical decline in older adults.

## Supplementary Information

Below is the link to the electronic supplementary material.

Supplementary Material 1

## Data Availability

All data supporting the findings of this study are available on request from the corresponding author.
