# Supplementary material for: Age-related differences in muscular coordination while performing elbow flexion and extension movements at different velocities
Source: Sci Rep. 2025 Sep 12;15:32486. doi: 10.1038/s41598-025-19126-z (PMC12432214; doi:10.1038/s41598-025-19126-z)
Supplement: Supplementary file 1 — Supplementary Material 1 [file 41598_2025_19126_MOESM1_ESM.docx]

**Age-related differences in muscular coordination while performing elbow flexion and extension movements at different velocities**

**Elisa Romero Avila^1,*^, Hannah Lena Siebers^2^, L. Cornelius Bollheimer^3^, Catherine Disselhorst-Klug^1^**

^1^Dept. of Rehabilitation & Prevention Engineering, Institute of Applied Medical Engineering,

RWTH Aachen University, Pauwelsstr. 20, 52074 Aachen, Germany.

^2^Experimental Orthopaedics and Trauma Surgery Teaching and Research Area, RWTH Aachen University, Pauwelsstr. 30, 52074 Aachen, Germany.

^3^Department of Geriatric Medicine, RWTH Aachen University, Pauwelsstr. 30, 52074 Aachen, Germany

*romero@ame.rwth-aachen.de

# **Supplementary Information**

**Table S1** p-values for statistical comparisons of muscular activation in the biceps brachii, brachioradialis and triceps brachii across the angular velocity categories during elbow flexion and extension. The significant values (p<0.05) are indicated in bold and reported separately for the young and older adult groups.

| **Muscle** | **Condition** | **Angular Velocity**  Category 1 | **Angular Velocity**  Category 2 | **Young**  *p-value* | **Older**  *p-value* |
| --- | --- | --- | --- | --- | --- |
| **Biceps brachii** | Flexion | 100-140°/s | 20 – 40°/s | **<0.001** | **0.002** |
|  |  |  | 40 – 60°/s | **<0.001** | **0.009** |
|  |  |  | 60 – 100°/s | **<0.001** | 0.072 |
|  | Extension | 100-140°/s | 20 – 40°/s | **<0.001** | **<0.001** |
|  |  |  | 40 – 60°/s | 0.060 | **0.003** |
|  |  |  | 60 – 100°/s | 0.214 | **0.022** |
| **Brachioradialis** | Flexion | 100-140°/s | 20 – 40°/s | **<0.001** | **<0.001** |
|  |  |  | 40 – 60°/s | **<0.001** | **0.003** |
|  |  |  | 60 – 100°/s | **<0.001** | **0.032** |
|  | Extension | 100-140°/s | 20 – 40°/s | **<0.001** | **<0.001** |
|  |  |  | 40 – 60°/s | 0.091 | **<0.001** |
|  |  |  | 60 – 100°/s | 0.073 | **0.001** |
| **Triceps brachii** | Flexion | 100-140°/s | 20 – 40°/s | **<0.001** | **0.011** |
|  |  |  | 40 – 60°/s | **<0.001** | **0.010** |
|  |  |  | 60 – 100°/s | **0.005** | 0.057 |
|  | Extension | 100-140°/s | 20 – 40°/s | 0.537 | **0.002** |
|  |  |  | 40 – 60°/s | 0.517 | **0.006** |
|  |  |  | 60 – 100°/s | 0.268 | 0.083 |
